# Limited clinical utility of a machine learning revision prediction model based on a national hip arthroscopy registry

**DOI:** 10.1007/s00167-022-07054-8

**Published:** 2022-08-10

**Authors:** R. Kyle Martin, Solvejg Wastvedt, Jeppe Lange, Ayoosh Pareek, Julian Wolfson, Bent Lund

**Affiliations:** 1 Department of Orthopaedic Surgery, University of Minnesota, 2512 South 7th Street, Suite R200, Minneapolis, MN 55455 USA; 2 Department of Orthopaedic Surgery, CentraCare, Saint Cloud, MN USA; 3 Division of Biostatistics, School of Public Health, University of Minnesota, Minneapolis, MN USA; 4 Department of Clinical Medicine, Aarhus University, Aarhus, Denmark; 5 CAAIR, Horsens Regional Hospital, Horsens, Denmark; 6 Department of Orthopedic Surgery, Mayo Clinic, Rochester, MN USA; 7 Department of Orthopedic Surgery, H-HiP, Horsens Regional Hospital, Horsens, Denmark

**Keywords:** Machine learning, Outcome prediction, Hip arthroscopy, Femoroacetabular impingement, Revision surgery

## Abstract

**Purpose:**

Accurate prediction of outcome following hip arthroscopy is challenging and machine learning has the potential to improve our predictive capability. The purpose of this study was to determine if machine learning analysis of the Danish Hip Arthroscopy Registry (DHAR) can develop a clinically meaningful calculator for predicting the probability of a patient undergoing subsequent revision surgery following primary hip arthroscopy.

**Methods:**

Machine learning analysis was performed on the DHAR. The primary outcome for the models was probability of revision hip arthroscopy within 1, 2, and/or 5 years after primary hip arthroscopy. Data were split randomly into training (75%) and test (25%) sets. Four models intended for these types of data were tested: Cox elastic net, random survival forest, gradient boosted regression (GBM), and super learner. These four models represent a range of approaches to statistical details like variable selection and model complexity. Model performance was assessed by calculating calibration and area under the curve (AUC). Analysis was performed using only variables available in the pre-operative clinical setting and then repeated to compare model performance using all variables available in the registry.

**Results:**

In total, 5581 patients were included for analysis. Average follow-up time or time-to-revision was 4.25 years (± 2.51) years and overall revision rate was 11%. All four models were generally well calibrated and demonstrated concordance in the moderate range when restricted to only pre-operative variables (0.62–0.67), and when considering all variables available in the registry (0.63–0.66). The 95% confidence intervals for model concordance were wide for both analyses, ranging from a low of 0.53 to a high of 0.75, indicating uncertainty about the true accuracy of the models.

**Conclusion:**

The association between pre-surgical factors and outcome following hip arthroscopy is complex. Machine learning analysis of the DHAR produced a model capable of predicting revision surgery risk following primary hip arthroscopy that demonstrated moderate accuracy but likely limited clinical usefulness. Prediction accuracy would benefit from enhanced data quality within the registry and this preliminary study holds promise for future model generation as the DHAR matures. Ongoing collection of high-quality data by the DHAR should enable improved patient-specific outcome prediction that is generalisable across the population.

**Level of evidence:**

Level III.

## Introduction

In 2003, Ganz et al. described femoroacetabular impingement (FAI) as one of the primary causes of hip osteoarthritis [10]. Over the last two decades, hip arthroscopy has been increasingly performed for the treatment of this intra-articular hip disorder along with cartilage and labral injuries [4, 7, 42]. As the annual number of procedures has increased, many studies have sought to evaluate the risk of undergoing a subsequent revision hip arthroscopy [1, 2, 5, 6, 9, 11, 12, 14, 17, 23, 28, 32, 34, 38]. Though these studies have identified several risk factors associated with revision surgery, the ability to translate these pre-operative factors into a specific risk score is poor. A clinical tool to estimate a patient’s individual risk of having subsequent revision hip arthroscopy would be a valuable adjunct for the surgeon to guide discussions regarding surgical decision-making and expectations.

Machine learning has the potential to improve the ability to estimate outcome at an individual level. Machine learning uses data to build flexible prediction and decision-making models without the need for researchers to pre-specify how predictors relate to each other and to the outcome of interest. Through analysis of large clinical datasets, machine learning models can identify factors associated with outcome and use these factors to formulate prospective predictive algorithms. The ideal database for clinically useful machine learning analysis is one that contains a large volume of patient data that is representative of a diverse portion of the population under evaluation. National registries represent a potentially strong data source which hold promise for the development of clinically impactful outcome prediction models due to the large volume of patients from multiple institutions and surgeons.

The Danish Hip Arthroscopy Registry (DHAR) has been prospectively collecting demographic, surgical, and outcome data since 2012. There are currently more than 6000 patients registered in the database who have undergone hip arthroscopy throughout Denmark. This national registry has yielded several clinically useful contributions to the orthopaedic literature [15, 26, 27, 29–31], and machine learning enables further analysis. The purpose of this study was to apply machine learning to the DHAR with the primary goal of developing a clinically useful algorithm capable of predicting subsequent revision hip arthroscopy. The hypothesis was that a resulting algorithm would be able to accurately estimate a patient’s risk of subsequent revision hip arthroscopy based on variables available in the pre-operative clinical setting. If successful, the resulting prediction model could be implemented in the clinic as an online calculator to guide discussions regarding surgical decision-making and outcome expectations at a patient-specific level.

## Materials and methods

At the time of data entry in the DHAR, all patients provide informed consent. The DHAR complies with all current national data protection legislature. Data management in the current study was performed confidentially according to Danish and European Union (EU) data protection rules, with all data de-identified prior to retrieval for analysis. As this was a register-based study, ethical approval was automatically waived according to national legislature.

### Transparent reporting

This manuscript was written in accordance with the Transparent Reporting of a multivariable prediction model for Individual Prognosis Or Diagnosis (TRIPOD) statement [3]. The TRIPOD statement represents recommendations for studies developing and/or validating prediction models. The goal of the TRIPOD statement is to improve the transparency of prediction model studies through full and clear information reporting and includes a 22-item checklist.

### Data preparation

Patients in the DHAR with primary hip arthroscopy dates between January 2012 and December 2020 were included. A full list of variables used in the analysis is shown Table 1a (pre-operative variables only) and 1b (intraoperative variables). Patients with previous surgery to the same hip were excluded to focus model prediction on patients undergoing primary hip arthroscopy for FAI. Additionally, a small number of patients with a history of Legg Calve Perthes, developmental dysplasia of the hip, avascular necrosis, slipped capital femoral epiphysis, or hip fracture were excluded to limit heterogeneity of the population and focus on surgical management of primary FAI. New variables were defined for type of previous injury to same hip (acetabular dysplasia, FAI), an indicator if the patient was missing any patient reported outcome variable, type of labral repair anchors (bioabsorbable, PEEK, all suture), number of anchors, type of knots, type of cartilage treatment (microfracture, fixation/resection), and type of other pathology found (adhesions, partial/full ligamentum teres rupture, synovitis, bursitis, calcified labrum, os acetabuli, loose bodies, other). The following variables were recoded: MRI performed (non-contrast, arthrogram) and Tönnis grade (Grades 0,1,2,3, and missing). Time to revision was calculated as number of months from primary hip arthroscopy to revision. For assessing concordance at specific follow-up times, patients with a revision at or prior to the time point were considered as having experienced the event.

**Table 1:** Characteristics of patients

a Characteristics of patients	
Variable*	Total *N* = 5581
Revision	603 (11%)
Time to revision or data current date (07–05-2021)	4.25 (2.51)
Age at surgery	38 (13)
Sex	
Female	3079 (55%)
Male	2502 (45%)
Hip pain	
Bilateral	1757 (31%)
Uni-lateral	3824 (69%)
Side	
Left	2526 (45%)
Right	3055 (55%)
Alpha angle	67 (14)
Missing	566
LCEA	31.0 (5.4)
Missing	200
Acetabular index	5.6 (4.3)
Missing	686
Tönnis grade	
Grade 0	1472 (70%)
Grade 1	595 (28%)
Grade 2	35 (1.7%)
Grade 3	3 (0.1%)
Missing	3476
Cross over sign	
Yes	1896 (46%)
No	2204 (54%)
Missing	1481
Ischial spine sign	
Yes	1518 (27%)
No	4063 (73%)
Posterior wall sign	
Yes	1011 (19%)
No	4294 (81%)
Missing	276
Joint space width	
< 2.0mm	12 (0.2%)
2.1–3.0mm	226 (4.0%)
< 3.1–4.0mm	1692 (30%)
> 4.0mm	3651 (65%)
Previous disorder/injury to same hip	361 (6.5%)
MRI performed	
Non contrast	923 (17%)
Arthrogram	3353 (62%)
None	1108 (21%)
Missing	197
Yearly volume: hospital	
> 100	2409 (43%)
1–12	51 (0.9%)
13–25	111 (2.0%)
26–50	866 (16%)
51–102	2143 (38%)
Missing	1
Yearly volume: surgeon	
> 100	2357 (42%)
1–12	197 (3.5%)
13–25	463 (8.3%)
26–50	1247 (22%)
51–100	1305 (23%)
Missing	12
Labral injury	5138 (92%)
PROM data: pre-operative VAS	42 (19)
Missing	2,277
PROM data: pre-operative EQ5D	0.65 (0.17)
Missing	2275
PROM data: pre-operative HAGOS PAIN	51 (19)
Missing	2,274
PROM data: pre-operative HAGOS SYMPTOMS	49 (18)
Missing	2,273
PROM data: pre-operative HAGOS ADL	53 (24)
Missing	2,274
PROM data: pre-operative HAGOS SPORT	36 (23)
Missing	2,273
PROM data: pre-operative HAGOS PA	22 (24)
Missing	2,274
PROM data: pre-operative HAGOS QOL	30 (16)
Missing	2,274
PROM data: pre-operative NRS REST	39 (25)
Missing	2277
PROM data: pre-operative NRS ACTIVITY	49 (27)
Missing	2278
PROM data: pre-operative HSAS	2.60 (1.96)
Missing	2269
Previous injury to same hip: Acetabular dysplasia	163 (2.9%)
Previous injury to same hip: FAI	193 (3.5%)

b Characteristics of patients (intraoperative variables	
Variable*	Total *N* = 5581
Operative duration	76 (33)
Missing	104
Traction time	46 (23)
Missing	106
Labral injury: type	
Bucket handle lesion	167 (3.3%)
Degenerative lesion	1022 (20%)
Fibrillation	123 (2.4%)
Labral flap tear	95 (1.9%)
Labral ossification	206 (4.0%)
Longitudinal injury at labral insertion site	3520 (69%)
Missing	448
Labral injury: treatment	
Full thickness labral resection	239 (4.7%)
Labral reconstruction	10 (0.2%)
Labral reinsertion	4237 (84%)
No procedures	6 (0.1%)
Partial labral resection	549 (11%)
Missing	540
Cartilage injury	4084 (73%)
Cartilage injury: size CAPUT	
< 1 cm2	395 (8.0%)
> 2 cm2	393 (8.0%)
1–2 cm2	564 (11%)
None	3562 (72%)
Missing	667
Cartilage injury: ICRS grade	
Grade 0	3538 (72%)
Grade 1	437 (8.9%)
Grade 2	600 (12%)
Grade 3	232 (4.7%)
Grade 4	107 (2.2%)
Missing	667
Cartilage injury: size ACETABULUM	
< 1 cm2	1627 (33%)
> 2 cm2	658 (13%)
1–2 cm2	2529 (51%)
None	100 (2.0%)
Missing	667
Cartilage injury: Beck's grade	
Grade 0	85 (1.7%)
Grade 1	623 (13%)
Grade 2	2253 (46%)
Grade 3	1449 (29%)
Grade 4	504 (10%)
Missing	667
Other pathology found	3339 (60%)
Bone resection	5368 (96%)
Bone resection: type	
CAM resection	907 (17%)
CAM resection, Rim Trim	3778 (70%)
Other	11 (0.2%)
Rim trim	671 (13%)
Missing	214
Extra-articular surgery	1626 (29%)
Peri-operative complications	554 (9.9%)
Type of anchors: bioabsorbable	66 (1.2%)
Type of anchors: PEEK	1507 (27%)
Type of anchors: all suture	997 (18%)
Number of anchors	1.16 (1.42)
Type of knots: knots	1662 (30%)
Type of knots: knotless	825 (15%)
Secondary anaesthesia: regional	803 (14%)
Secondary anaesthesia: local	3189 (57%)
Secondary anaesthesia: peri-articular	2935 (53%)
Cartilage treatment: microfracture	242 (5.9%)
Missing	1503
Cartilage treatment: fixation or resection	
Cartilage fixation	19 (0.5%)
None	65 (1.6%)
Resection/debridement	3994 (98%)
Missing	1,503
Other pathology: adhesions	18 (0.5%)
Missing	2256
Other pathology: partial ligamentum teres rupture	135 (4.1%)
Missing	2256
Other pathology: full ligamentum teres rupture	195 (5.9%)
Missing	2,256
Other pathology: synovitis	3012 (91%)
Missing	2256
Other pathology: bursitis	21 (0.6%)
Missing	2256
Other pathology: calcified labrum	276 (8.3%)
Missing	2256
Other pathology: Os Acetabuli	272 (8.2%)
Missing	2256
Other pathology: loose bodies	89 (2.7%)
Missing	2256
Other pathology: other	351 (11%)
Missing	2256

### Machine learning modelling

The cleaned data were split randomly into training (75%) and test (25%) sets for model fitting and evaluation, respectively. The primary outcome for the models was probability of revision hip arthroscopy within 1, 2, and/or 5 years after primary hip arthroscopy. This approach utilises a survival-analysis temporal framing structure [25] and the program R (version: 4.1.1, R Core Team 2021, R Foundation for Statistical Computing, Vienna, Austria) was used to fit and evaluate several models adapted for censored, time-to-event data. “Censoring” refers to the fact that at any given time, complete information is not known for all the patients in the registry. For example, if a patient has two years of follow-up after primary surgery with no revision, we do not know if or when that patient will go on to have a revision. Models adapted for censored data allow use of the partial information contained in these censored observations while accounting for the incompleteness.

The following four machine learning models were used: Cox elastic net, random survival forest, gradient boosted regression (GBM), and super learner. The Cox elastic net is a penalised, semi-parametric regression model that selects a subset of the predictors for inclusion in the model. “Elastic net” refers to the combination of L1 and L2 penalties used to shrink model coefficients toward zero [35]. The random survival forest is an adaptation of the popular tree-based random forest method for censored data. It uses all predictors and is nonparametric, meaning it does not require specification of the model structure [16]. The GBM is also tree based and nonparametric. It iteratively improves the model fit using all predictors [8]. The super learner is an “ensemble” technique that averages over model fits from several different types of models for an even more flexible approach [24]. Our super learner combined all the other three model types: Cox elastic net, random survival forest, and GBM.

**Fig. 1:**
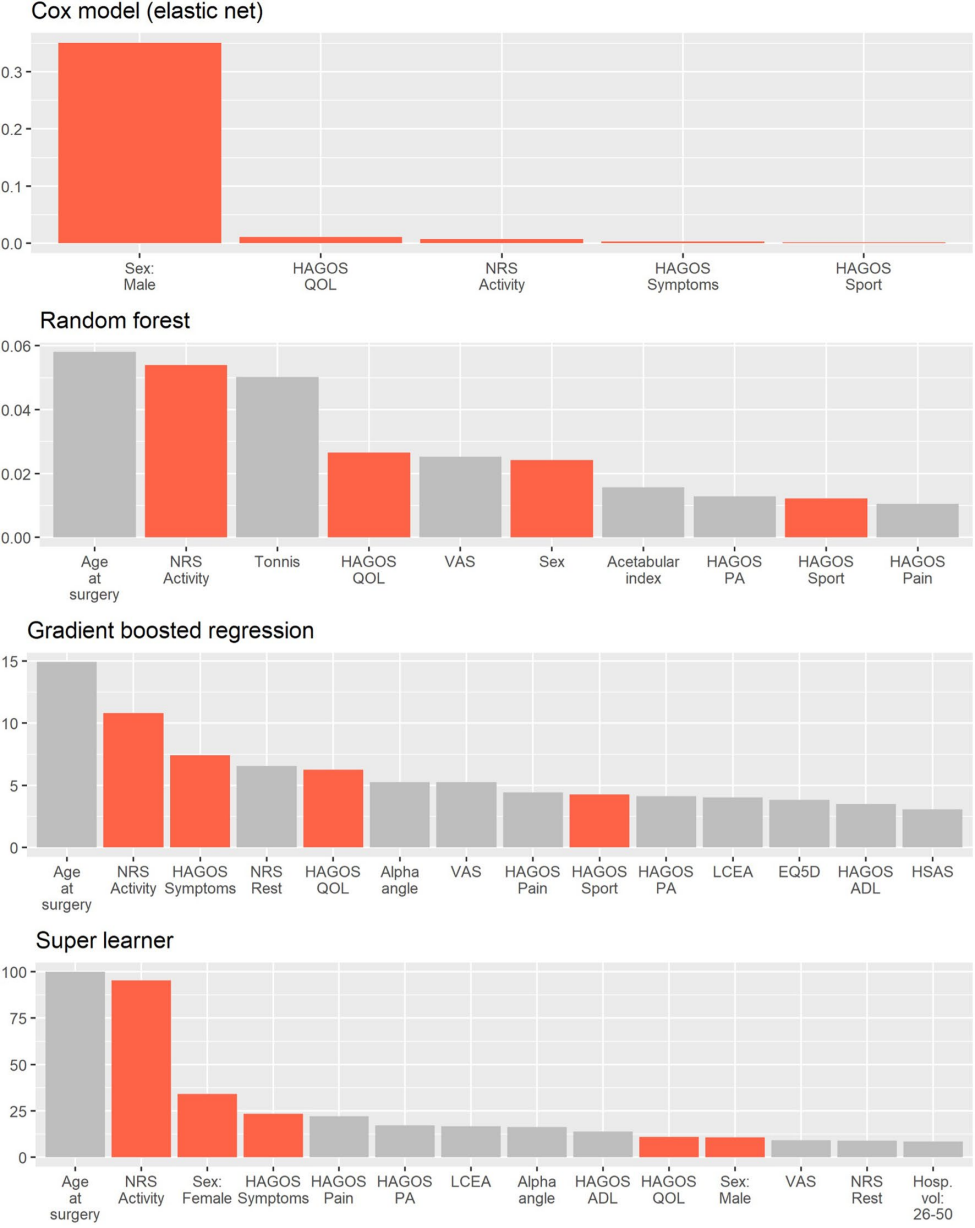
Variable importance. The four plots show relative feature importance in each of the machine learning models. The highlighted bars indicate features selected into the Cox model. Random forest, gradient boosted (GBM), and super learner plots show features in the top half by importance score, for readability. Feature importance is measured on a different scale for each model, and thus only rankings of features, rather than scores, should be compared among the models. The Cox model measures feature importance by absolute effect size. The random forest and super learner models use permutation-based importance, which measures the relative change in model performance upon randomly permuting values of the given feature. The GBM uses difference in error rate were the feature to be removed, normalised to sum to 100

The Cox elastic net model (package *glmnet*, alpha value 0.9, lambda value selected via cross-validation) was fit to the data and predictors with non-zero coefficients were retained, shown in the top panel of Fig. 1. The random survival forest, GBM, and super learners were fit using a grid search method to arrive at hyperparameters (package *MachineShop*). The grid search method compares all possible combinations of a given set of hyperparameters to find the best fit based on a specified performance metric, for which the C-Index was used, as described below. The random survival forest (package *randomForestSRC*) used 1000 trees, a minimum node size of 200, and 10 variables tried per split. The GBM (package *gbm)* used 1000 trees, and interaction depth of 3, minimum node size of 100, and shrinkage of 0.01. The super learner model (*SuperModel* function, package *MachineShop*) combined the three previous models with the specified hyperparameters.

Each of the machine learning models was fit using two different sets of predictors: all predictors, and all predictors excluding intraoperative variables (Table 1a). The two separate analyses allowed for comparison of model performance given only variables available in the pre-operative setting versus a model considering all variables available after surgical intervention.

### Model evaluation

Performance measures adapted for censored data were used to evaluate the four models on survival probabilities calculated for the hold-out test set. A measure of model concordance adapted for censored data, Harrell’s C-Index, was used at 1-, 2-, and 5-year follow-up times. The C-Index computes the proportion of pairs of observations in which predicted survival probability ranking corresponds to actual ranking [13]. It is a generalisation of the common area under the Receiver Operating Characteristics curve (AUC) metric for censored data and, as with AUC, ranges from 0 to 1 with 1 indicating perfect concordance and 0.5 representing random chance. Concordance is a measure of the model’s ability to differentiate between patients who do and do not experience the event. A model is said to have perfect concordance if the predicted risks for all individuals who experience the outcome are higher than those for all those who do not. Most clinically useful prediction models have a concordance in the 0.65–0.8 range [41]. Calibration which was adapted for censored data was also calculated. Calibration measures the accuracy of the predicted probabilities by comparing actual to expected outcomes. For this purpose, a version of the Hosmer–Lemeshow statistic intended for censored data was used. The statistic sums average misclassification in predicted risk quintiles and converts the sum into a chi-squared statistic [37]. Larger values of the calibration statistic indicate worse accuracy and produce smaller p-values. Statistical significance of the calibration statistic means we reject the null hypothesis of perfect calibration. Each of these performance metrics was calculated separately for models trained using the full set of predictors and pre-operative variables only.

### Missing data

Because of high rates of missing data (Table 1) on some variables used for prediction, imputation was performed on the cleaned data prior to analysis. The imputation was performed via random forest (function *missForest* in package *missForest*) to arrive at a single imputed data set for each of the training and test data. The random forest imputation method trains a random forest on the observed data and uses it to predict imputed values for missing data [36]. To avoid leakage between the training and test data, the forest was trained on only the training observed data and was then used to predict for both training and test sets. All models were fit and evaluated on the imputed training and test sets, respectively. Imputation was performed separately for the two analyses described above (pre-operative only and all variables). In each case, only the predictor variables included in the specified analysis were used in imputation.

## Results

### Data characteristics

After data cleaning, 5581 patients were included in the analysis (713 patients excluded for previous hip surgery, 16 more patients excluded based on type of previous injury to same hip). Table 1 describes the characteristics of the population at the time of primary hip arthroscopy and lists all predictor variables considered in the analysis. Of the patients included after data cleaning, 603 (11%) underwent revision surgery, during an average follow-up time of 4.25 years (SD 2.51). The population was predominantly female (3079 patients; 55%), the average alpha angle was 67 (SD 14), average Tönnis grade was 0, and the majority had uni-lateral hip pain (3824 patients; 69%). Table 2 describes the number of patients who experiences revision at or before 1, 2, and 5 years post primary surgery as well as the number with complete follow-up but no revision, and the number censored before the follow-up time.

**Table 2:** Description of censoring

Follow-up time (years)	Revision	Complete follow-up, no revision	Censored
1	216 (3.9%)	5036 (90.2%)	329 (5.9%)
2	448 (8%)	4167 (74.7%)	966 (17.3%)
5	576 (10.3%)	2286 (41%)	2719 (48.7%)

### Machine learning model performance

The four models exhibited concordance in the moderate range across the follow-up times when restricted to only pre-operative variables (0.62–0.67) and exhibited similar concordance when using all variables (Tables 3, 4). The 95% confidence intervals for model concordance were wide for both analyses, ranging from a low of 0.53 to a high of 0.75, indicating uncertainty about the true concordance of the models. The random survival forest and GBM had a slight edge over the other two models in terms of concordance at 1-, 2-, and 5-year follow-up times using only pre-operative variables. The GBM had the best concordance of the models for the analysis using all variables. In general, the models were well calibrated, with only the random survival forest showing evidence of mis-calibration at 1 year (*p* value less than 0.01) and slight evidence of mis-calibration at 5 years (*p* value between 0.01 and 0.05) for the analysis restricted to pre-operative variables. For the analysis using all variables, only the Cox elastic net model showed evidence of mis-calibration at 1 year and slight evidence of mis-calibration at 5 years.

**Table 3:** Model performance

Outcome	Model	Concordance	Concordance 95% CI	Calibration statistic	Calibration *p* value
1 year	Cox model (elastic net)	0.63	(0.54, 0.71)	3.41	0.333
	Random forest	0.67	(0.58, 0.75)	13.05	0.005
	Gradient boosted regression	0.67	(0.58, 0.75)	3.66	0.3
	Super learner	0.64	(0.55, 0.72)	2.32	0.508
2 years	Cox model (elastic net)	0.62	(0.54, 0.71)	2.19	0.533
	Random forest	0.65	(0.57, 0.73)	2.49	0.477
	Gradient boosted regression	0.66	(0.57, 0.74)	0.28	0.963
	Super learner	0.63	(0.55, 0.72)	1.5	0.682
5 years	Cox model (elastic net)	0.62	(0.53, 0.71)	1.49	0.684
	Random forest	0.65	(0.56, 0.73)	7.94	0.047
	Gradient boosted regression	0.65	(0.57, 0.74)	1.45	0.695
	Super learner	0.63	(0.54, 0.71)	3.59	0.31

**Table 4:** Model performance – all variables

Outcome	Model	Concordance	Concordance 95% CI	Calibration statistic	Calibration *p*-value
1 year	Cox model (elastic net)	0.64	(0.55, 0.72)	21.28	< 0.001
	Random forest	0.64	(0.55, 0.73)	3.43	0.329
	Gradient boosted regression	0.66	(0.57, 0.74)	4.84	0.184
	Super learner	0.64	(0.55, 0.72)	6.89	0.075
2 years	Cox model (elastic net)	0.63	(0.55, 0.72)	1.81	0.612
	Random forest	0.63	(0.54, 0.72)	2.78	0.428
	Gradient boosted regression	0.65	(0.57, 0.74)	2.67	0.445
	Super learner	0.63	(0.55, 0.72)	1.98	0.577
5 years	Cox model (elastic net)	0.63	(0.55, 0.72)	8.3	0.04
	Random forest	0.63	(0.54, 0.72)	3.1	0.376
	Gradient boosted regression	0.65	(0.56, 0.74)	1.13	0.771
	Super learner	0.63	(0.54, 0.72)	2.47	0.481

### Factors predicting risk of revision surgery

Variables with non-zero coefficients in the pre-operative variable Cox elastic net model were, in order of importance: sex, pre-operative HAGOS Quality of Life score, pre-operative NRS Activity score, and pre-operative HAGOS Symptoms and Sport scores. The relative importance of these variables for predicting probability of revision surgery is shown in the top panel of Fig. 1, where the size of each bar corresponds to the absolute value of the variable’s effect size. Variables in the top third by importance for the other three pre-operative variable models also included pre-operative HAGOS scores, pre-operative NRS Activity score, and sex (random survival forest and super learner). However, age at surgery was the most important variable for these three models (Fig. 1, bottom three panels). The random survival forest and super learner models use permutation-based variable importance, which measures importance as the relative change in model performance upon randomly permuting values of the given variable. The GBM quantifies importance as difference in error rate were the variable to be removed.

## Discussion

The most important finding of this study is that while machine learning analysis of a national hip arthroscopy registry enabled the development of algorithms capable of predicting subsequent revision surgery, the clinical utility of these models is likely limited. Analysis was performed using only variables that would be available in the pre-operative setting and again using the full data set. Both scenarios resulted in well-calibrated models with moderate concordance, but also with wide confidence intervals that approached random chance. Overall, the analysis was limited by a substantial proportion of missing data but encourages optimism for future models if data collection can be improved.

Machine learning represents an approach to health care research that is increasingly being applied to analyse large orthopaedic databases. The main advantage of machine learning relates to the ability of the technique to realise complex associations and relationships within large datasets. With minimal direct human programming, these models can “learn” which factors are associated with a specified outcome and can then create an algorithm with the goal of accurate outcome prediction. The most common machine learning applications in orthopaedic surgery involve clinical prediction modelling and automated image interpretation. It is anticipated that machine learning models will serve as a valuable adjunct for clinicians in the future, guiding clinical discussions at a patient-specific level.

Within the field of hip arthroscopy several studies have now been performed that seek to predict patient-specific outcome following the procedure. Most have focused on patient reported outcome, with Kunze et al. analysing single-surgeon data to predict multiple post-operative endpoints based on different outcome measuring tools [19–22]. The prediction of subsequent surgery following hip arthroscopy has also been performed by Haeberle et al. based on another single-surgeon database of over 3000 patients [11]. With their study, Haeberle et al. achieved an AUC of 0.77 ± 0.08 for predicting a patient’s risk of subsequent revision hip arthroscopy. These early studies show promise for clinical usefulness of hip arthroscopy prediction models but are of uncertain real-world applicability due to the single-surgeon nature of the databases and lack of external validation.

This study represents the first national registry-based machine learning model for hip arthroscopy outcome prediction. The goal of the present study was to develop an accurate model based on pre-operative variables that could provide a risk estimate for subsequent hip arthroscopy at a patient-specific level. This would allow a surgeon to input their patient’s data into a prediction calculator during the initial patient encounter and estimate that patient’s individual revision surgery risk. This information could then guide expectations and the surgical with the patient. While the results of this study did demonstrate the ability to predict revision surgery with reasonable accuracy, the wide discrimination confidence interval likely limits the clinical utility of the algorithms.

There are some possible explanations for the inferior model performance of the present study relative to the revision hip arthroscopy model developed by Haeberle et al. [11]. Although overall compliance with the DHAR is between 78–97% annually [43], the completeness of the data limits the ability of the models to accurately predict outcome. This is partly due to the fact that as the DHAR evolved from the initial stages through to the present version, some variables were added, removed, or modified which contributes to the data inconsistency. Variance within the DHAR is also expected, given the multiple-surgeon nature of the registry while the single-surgeon institutional registry likely benefits from more overall consistency. As more patients are enrolled and the data collection stability improves, it is anticipated that future machine learning analysis of the DHAR may yield improved prediction accuracy.

The variables recorded in the DHAR itself may also limit the ability of machine learning analysis to develop useful risk prediction models. The multiple factors included in the register were chosen by the founding surgeons as they were felt to be the most relevant based on current literature. It is possible that some factors not currently included in the DHAR may in fact be more strongly associated with outcome and thus, their exclusion may bias the models toward suboptimal performance. Future analysis may clarify this limitation and the advancements of other machine learning techniques such as computer vision [18] and natural language processing [39, 40] may make register-based data collection both simpler and more comprehensive.

Substantial missing data represent the main limitation of this study while there are other limitations to also consider. First, four common machine learning models that represent various approaches to variable selection and model complexity were selected for data analysis, but it is possible that a model that was not considered may have performed better. Second, the analysis included all variables in the DHAR but there may be other factors associated with the risk of subsequent surgery which are not included in the registry and therefore not considered in our models. Some examples of factors that may be relevant for outcome prediction include clinical examination findings, rehabilitation details, or raw imaging data files. The main concern regarding clinical applicability of this study lies in the accuracy of the model, with concordance limited by a wide confidence interval approaching random chance. Additionally, the ability to pre-operatively predict who is at risk of subsequent revision hip arthroscopy is likely limited by the endpoint itself. That is, a common reason that is often cited for revision surgery is residual CAM deformity—a factor that is not known in the pre-operative setting [2, 12, 33, 38].

Although the results from this preliminary study are not suitable for immediate clinical application, it should serve as a baseline for future outcome prediction studies applying machine learning to large hip arthroscopy datasets. Additionally, there is optimism regarding the future development of patient-specific revision risk estimation if data collection can be improved. Accurate prediction of outcome using machine learning relies on both data quantity and quality. As a national registry, the DHAR will naturally continue to grow the quantity of data collected over time as all hip arthroscopy procedures performed in Denmark are captured. Data quality is more challenging to improve upon. Overcoming bias related to the surgeon-selected nature of the variables currently collected by the registry will require ongoing critical assessment over time and emerging technology like natural language processing for data collection may enable the identification of additional variables that may influence outcome. Another way to potentially improve machine learning driven outcome prediction is through the creation of an international hip arthroscopy register or collaboration between national registers. International collaboration would require a pre-determined definition of a minimum common dataset across registers but would greatly improve predictive power through data sharing. Resulting algorithms could then be implemented into clinical practice to guide outcome expectations and discussions around surgical decision-making in the pre-surgical setting.

## Conclusion

The association between pre-surgical factors and outcome following hip arthroscopy is complex. Machine learning analysis of the DHAR produced a model capable of predicting revision surgery risk following primary hip arthroscopy that demonstrated moderate accuracy but likely limited clinical usefulness. Prediction accuracy would benefit from enhanced data quality within the registry and this preliminary study holds promise for future model generation as the DHAR matures. Ongoing collection of high-quality data by the DHAR should enable improved patient-specific outcome prediction that is generalisable across the population.
